# Air travel with pneumocephalus: a systematic review

**DOI:** 10.1007/s00701-022-05297-5

**Published:** 2022-07-06

**Authors:** Oliver Bichsel, Annalisa Hauck, Markus Oertel

**Affiliations:** 1 Department of Neurosurgery, University Hospital Zurich, University of Zurich, Zurich, Switzerland; 2 Clinical Neuroscience Centre, University Hospital Zurich, University of Zurich, Zurich, Switzerland; 3 Department of Health Sciences and Technology, ETH Zurich, Zurich, Switzerland; 4 Medical Sciences Division, University of Oxford, Oxford, UK

**Keywords:** Pneumocephalus, Intracranial air, Intracranial gas, Air travel, Flying

## Abstract

**Introduction:**

Concerns arise when patients with pneumocephalus engage in air travel. How hypobaric cabin pressure affects intracranial air is largely unclear. A widespread concern is that the intracranial volume could relevantly expand during flight and lead to elevated intracranial pressure. The aim of this systematic review was to identify and summarise models and case reports with confirmed pre-flight pneumocephalus.

**Methods:**

The terms (pneumocephalus OR intracranial air) AND (flying OR fly OR travel OR air transport OR aircraft) were used to search the database PubMed on 30 November 2021. This search returned 144 results. To be included, a paper needed to fulfil each of the following criteria: (i) peer-reviewed publication of case reports, surveys, simulations or laboratory experiments that focussed on air travel with pre-existing pneumocephalus; (ii) available in full text.

**Results:**

Thirteen studies met the inclusion criteria after title or abstract screening. We additionally identified five more articles when reviewing the references. A notion that repeatedly surfaced is that any air contained within the neurocranium increases in volume at higher altitude, much like any extracranial gas, potentially resulting in tension pneumocephalus or increased intracranial pressure.

**Discussion:**

Relatively conservative thresholds for patients flying with pneumocephalus are suggested based on models where the intracranial air equilibrates with cabin pressure, although intracranial air in a confined space would be surrounded by the intracranial pressure. There is a discrepancy between the models and case presentations in that we found no reports of permanent or transient decompensation secondary to a pre-existing pneumocephalus during air travel. Nevertheless, the quality of examination varies and clinicians might tend to refrain from reporting adverse events. We identified a persistent extracranial to intracranial fistulous process in multiple cases with newly diagnosed pneumocephalus after flight. Finally, we summarised management principles to avoid complications from pneumocephalus during air travel and argue that a patient-specific understanding of the pathophysiology and time course of the pneumocephalus are potentially more important than its volume.

## Introduction

Pneumocephalus refers to the presence of gas within the neurocranium. Aetiologies of pneumocephalus are numerous and include neurosurgical procedures, trauma, congenital or erosive skull defects, and infections with gas-producing organisms.

Great concerns arise when patients with pneumocephalus must or wish to engage in air travel. These concerns primarily arise from the potential effect changing cabin pressure could have on the intracranial air volume and ultimately on intracranial pressure. Although commercial airplanes usually cruise at altitudes of 7000–13,000 m above sea level, the passenger cabin is pressurised to an altitude of 1500–2500 m above sea level.

Boyle’s law states that, given a constant mass and temperature of an ideal gas, pressure is inversely proportional to the volume ($P\propto 1/V$). Thus, a closed air-filled balloon in an aircraft, for instance, would tend to increase in the hypobaric environment at cruising altitude.

It is unclear how hypobaric cabin pressure affects intracranial air and this incertitude is reflected in inconsistent recommendations towards patients with pneumocephalus [1, 18]. The main concern regarding flying postintracranial surgery is that any residual air in the cranium may potentially expand converting a simple pneumocephalus into a life-threatening tension pneumocephalus [1]. Given the current lack of clear clinical guidelines, a definite recommendation as to which patients with pneumocephalus can travel safely is not possible. Nevertheless, correctly identifying patients with pneumocephalus that are at increased risk during flight could help prevent the occurrence of increased mass effect by postponing the flight or establishing management principles in case air travel cannot be delayed.

The aim of this article is to identify peer-reviewed publications that focussed on flying with pneumocephalus in order to summarise the findings from case reports with confirmed pre-flight pneumocephalus, simulations or laboratory experiments in a systematic review.

## Methods

This systematic review was designed using the PRISMA guidelines [16]. The terms (pneumocephalus OR intracranial air) AND (flying OR fly OR travel OR air transport OR aircraft) were used to search the database PubMed from its respective commencement until 30 November 2021. No earliest date was set before which studies would be excluded. An English language filter was then applied to the search results.

The titles and abstracts of the articles identified by this search were reviewed to determine whether they fulfilled inclusion criteria. Articles that possibly fulfilled inclusion criteria were retrieved and read in full text. To be included, a paper needed to fulfil each of the following criteria: (i) peer-reviewed publication of case reports, surveys, simulations or laboratory experiments that focussed on air travel with pre-existing pneumocephalus; (ii) available in full text. A search of the reference lists of the included articles was conducted to assess for further studies that may fulfil the inclusion criteria.

## Results

The initial search returned a total of 144 results. After title or abstract review, thirteen articles fulfilling the inclusion criteria were viewed in full text. The reference lists of these articles were searched for further studies that may fulfil the inclusion criteria, yielding five additional studies to be included. Thus, 18 publications met all inclusion criteria and were finally included.

### Case presentations

Donovan et al. [9] looked retrospectively at the air transport of 21 combat casualties with confirmed pneumocephalus (volumes ranging from 0.6 to 42.7 ml). None of the patients suffered a temporary or permanent neurological deficit (and in the three patients with invasive monitoring, there was no evidence of sustained intracranial pressure elevations), suggesting that pneumocephalus per se is no contraindication for flying [1].

A patient with an osteoma invading the frontal sinus and secondary periorbital emphysema, pneumocephalus and cerebrospinal fluid leak was safely air-transported by a commercial airline to a speciality care facility [17]. Interestingly, the patient underwent additional postoperative evaluations including hypobaric testing up to a maximum of 5486 m at 6 months after the surgery before later successfully returning to his aviation duties. Willson et al. [20] reported on an uneventful 45-min commercial air travel of a patient 1 day after the introduction of intracranial air during functional endoscopic sinus surgery.

A patient with severe obtundation from tension pneumocephalus due to a fracture of the cribriform plate after endoscopic sinus surgery for chronic sinusitis on the preceding day was safely flown by fixed wing aircraft for urgent neurosurgical decompression and skull base repair at an urban centre [3]. During flight, the cabin pressure was maintained at the atmospheric pressure of the departing airport and the patient was receiving 10 l/min of oxygen via a non-pressurised, non-rebreather mask in a flat supine position.

Of note, a patient with a small anterior table defect as well as a minimally displaced posterior table fracture of the frontal sinus underwent shrapnel removal, primary closure and nasal packing at a combat support hospital before evacuation by air [11]. Despite the minimally displaced posterior table of the frontal sinus, the CT revealed no evidence of intracranial air, while, nevertheless, there was air ‘mushrooming’ out of the anterior table defect and elevating the scalp tissue. During the endoscopic visualisation of the posterior table, there was no gross leak of cerebrospinal fluid with Valsalva manoeuvre from the 5–8-mm oval-shaped fragment without mucosal covering that was retrodisplaced approximately 2–3 mm. The patient then returned by low-level flight with an angiocatheter placed into the anterior table defect and allowed to vent to air.

### Simulations and laboratory experiments

The hydrodynamics of a pneumocephalus patient during flight was investigated in an experimental setup using an acrylic box (skull), air-filled balloon (intracranial air), water-filled balloon (cerebrospinal fluid and blood) and agarose gel (brain) all placed in a custom-made pressure chamber [14]. An intracranial air volume of 20 ml and an initial intracranial pressure of 15 mm Hg were recommended as conservative thresholds that are required for safe air travel among pneumocephalus patients.

Two simulation studies used the same model for the nonlinear transformation of air volume expansion into intracranial pressure [2, 5]. The model is built on the assumption that intracranial gas volume expands (given unchanged temperature) at decreased cruising cabin pressure, which in turn leads to an increase in intracranial pressure as long as the dura mater and/or calvarium is intact [5]. The simulation with this model suggested that intracranial air volumes above 11 ml could result in intracranial hypertension during the drop in cabin pressure [5]. Moreover, the increase in ICP was also found to depend on the rate of change in cabin pressure [2, 5]. The computer simulations by Andersson et al. modelled that the usual changes in cabin pressure may increase intracranial air volume by approximately 30% [2].

## Discussion

The presence of pneumocephalus causes great uncertainty and safety concerns when patients with intracranial air inclusions must or wish to engage in air travel. These concerns mostly stem from the potential effect changing cabin pressure could have on intracranial air and consecutively on intracranial pressure. A systematic review was conducted to gain and summarise further information from case reports and series, surveys, laboratory experiments and simulations concerning patients with pneumocephalus engaging in air travel.

### Safety concerns fuelled by models

A notion that repeatedly surfaced in this body of literature is that any air or gas contained within the neurocranium expands at higher altitude, comparable to extracranial gas, potentially resulting in increased intracranial pressure or even tension pneumocephalus.

The model in both simulation studies [2, 5] assumes that the pressure inside the intracranial air equilibrates with the surrounding cabin pressure [14]. In other words, the model assumes the intracranial gas to expand until its pressure is equivalent to that of the cabin [14]. The underlying assumption of the model has been criticised, as the rigidity of the surrounding brain is not factored in [14]. In contrast, we argue that an intact physical barrier, consisting inter alia of dura and cranium, creates, under normal circumstances, a positive pressure environment relative to the atmosphere (i.e. intracranial pressure). Given a rigid cranium and an intact physical barrier, changes in the atmospheric pressure do not directly affect the intracranial pressure and therefore also not any intracranial air that is completely surrounded by the intracranial pressure. Rather, under these circumstances, changes in cabin pressure *indirectly* affect intracranial pressure, and intracranial air is only then affected by a changing intracranial pressure. Moreover, there are compensatory mechanisms (cerebrospinal fluid management, arterial pressure, venous return etc.) and the expansion of the intracranial air would be limited by the tension of surrounding tissue and fluids as well as a possibly increasing intracranial pressure (given the expansion of the air inclusion within a closed-compartment).

In the only laboratory experiment conducted so far to investigate pneumocephalus during flight, four grooves were cut into the lid of the acrylic box (skull model) in order to allow the intracranial air to react with the changes in air pressure inside the pressure chamber [14].

Based on their underlying assumptions, the models so far reported in the literature suggest overly conservative thresholds for patients flying with pneumocephalus.

### Discrepancy between safety concerns and case presentations

The concern that intracranial air relevantly expands with increasing altitude is widespread [1]. Nevertheless, older reviews [12, 18] as well as this systematic review found no reports of decompensation of either permanent or transient nature secondary to a pre-existing pneumocephalus during air travel.

The case series by Donovan et al. [9] is reassuring, as the air-evacuation of 21 combat casualties with confirmed pneumocephalus (volumes ranging from 0.6 to 42.7 ml) was without complications and the intracranial pressure monitoring, which was performed in three patients, without sustained elevations [1]. Moreover, we found no indication of transient dangerous intracranial pressure elevations in this case series. Furthermore, a patient with an osteoma invading the frontal sinus and secondary periorbital emphysema, pneumocephalus and cerebrospinal fluid leak was safely transported by a commercial airline to a speciality care facility [17]. While we found no case reports of decompensation secondary to a pre-existing pneumocephalus during air travel, the quality of examination varies and clinicians might tend to refrain from reporting such adverse events.

### Caution in patients with persistent extracranial to intracranial fistulous processes

While all case presentations fulfilling the inclusion criteria of this review showed no decompensation of the pneumocephalus, we would like to advise caution to clinicians regarding patients with a potential extracranial to intracranial fistula. Complications with intracranial air inclusions after flight have been reported in patients without a pre-flight diagnosis of pneumocephalus. A patient with a likely persistent intracranial to extracranial fistulous tract after parietotemporal craniectomy, subsequent debridement due to infection and still persistent complex dehisced scalp wound was found unarousable and with dramatic pneumocephalus after air travel [4]. One patient presented with Mount Fuji sign following cervical epidural injection and subsequent air travel [19]. Since no imaging was performed prior to the flight, it remains unclear how severe the pneumocephalus was prior to air travel and the authors suggested an inadvertent dural puncture as the aetiology of the pneumocephalus.

Another impending dramatic in-flight evolution of pneumocephalus with an extracranial to intracranial fistulous tract was presumably averted by maintaining the atmospheric pressure of the departing airport and by the patient receiving 10 l/min oxygen via a non-pressurised, non-rebreather mask in a flat supine position [3].

One patient suffered multiple facial fractures and a dural tear on his way to the airport and without neurologic deterioration in-flight eventually presented to the emergency department with an extensive pneumocephalus [6]. The CT scan 7 days after surgery, including cranialisation of the frontal and ethmoidal sinuses as well as repair of the dural tear using a vascularised pericranial flap, showed minimal pneumocephalus. The patient was allowed to fly home again and has since made a complete recovery. Fistulous processes can also appear after barotrauma from air-filled sinuses or mastoid air cells [6].

A patient with a minimally displaced posterior table fracture of the frontal sinus showed no intracranial air inclusion after evacuation by air [11]. Subsequent endoscopic visualisation showed no evidence of gross leak of cerebrospinal fluid with Valsalva manoeuvre, suggesting that there was no extracranial to intracranial fistulous defect.

Complications with intracranial air inclusions after travel by air have been solely reported in patients with persistent extracranial to intracranial fistulous processes, regardless of a pre-flight diagnosis of pneumocephalus. Potential mechanisms for the generation of pneumocephalus with extracranial to intracranial fistulous processes include valve mechanisms, Valsalva manoeuvres or the presence of a cerebrospinal fluid leak [15], which in turn creates a pressure gradient that can lead to an influx of air [7].

### Management principles to avoid complications from pneumocephalus during flight

Specific precautions to be observed to avoid complications from pneumocephalus during flight can be suggested but ultimately lack conclusive data [3]. A low-level flight or maintenance of ground-level cabin pressure during the flight might be considered to avoid hypobaric atmosphere pressures. If unavailable, lower rates of change in cabin pressure should be preferred. Other suggestions include positioning such that cerebrospinal fluid leak is minimised [3], supplementary oxygen administration in order to facilitate the diffusion of nitrogen from the intracranial air to the surrounding cerebral tissue [8, 10], preflight decongestants [11] and avoidance of Valsalva manoeuvres. Furthermore, in ventilated patients, hypoventilation and carbon dioxide retention resulting in vasodilation and increased ICP should be avoided. Recent pre-flight imaging is indicated to assess for the presence of any intracranial air volume or potential extracranial to intracranial fistulous processes especially in constellations that predispose to these phenomena and especially when there are unexplained neurological findings. An understanding of the patient-specific pathophysiology behind the pneumocephalus and its time course might be more important than its volume. We argue that as long as the intracranial air is confined and completely surrounded by the intracranial pressure, the atmospheric pressure does not have a *direct* effect on the intracranial air.

Besides hypobaric conditions, other constellations should be considered when flying with pneumocephalus. For instance, stresses caused by accelerations (including changing g-forces), noises or hypoxaemia because of decreased partial pressure of oxygen at higher altitudes are relevant factors [13]. Hypoxaemia in turn could result in increased cerebral blood flow for compensation, which potentially creates an increased risk of increased intracranial pressure and haemorrhagic or ischaemic events. In high-volume pneumocephalus, the surrounding brain tissue might also be more susceptible to the environmental stresses presented during flight. Concerns regarding complications away from home and less favourable treatment conditions might also arise [1].

### Limitations

This review is limited by the exclusion of non-English articles. No formal assessment of publication bias was conducted. The overall sparse literature on this topic, the paucity of case reports and the heterogeneous ways of reporting limit generalising the results of this review, drawing final conclusions and making concrete recommendations. Moreover, clinicians might tend to refrain from reporting adverse events giving rise to a possible bias and the reports do not present enough basic data enabling a good scientific evaluation.

### Outlook

Given the current lack of clear clinical guidelines, an informed decision as to which patients with pneumocephalus can travel safely is still not possible. Rigorous reporting of future case reports and in vivo recordings of intracranial pressure during flight will help addressing this important clinical question and spark discussions to prevent repetition of errors leading to complications. In addition, more accurate simulation studies and laboratory experiments are required to better understand what occurs to intracranial air accumulations in closed intracranial compartments. Correctly identifying patients with pneumocephalus that are at increased risk during flight could help prevent the occurrence of increased mass effect by postponing the flight or establishing management principles in case air travel cannot be delayed.

## Conclusion

Concerns arise when patients with intracranial air inclusions engage in air travel, mainly due to an uncertainty of how hypobaric cabin pressure affects intracranial air. The notion that intracranial air expands much like extracranial gas seems overrated and fuelled by results from models and experiments with assumptions and conditions that can be discussed. Along this line, we found no case reports of decompensation secondary to a pre-existing pneumocephalus during air travel. Nevertheless, the quality of examination varies and clinicians might tend to refrain from reporting such adverse events. A red flag in the form of a persistent extracranial to intracranial fistulous process was identified to be the cause for complications with intracranial air inclusions in multiple cases without a pre-flight diagnosis of pneumocephalus. A patient-specific understanding of the pathophysiology behind the pneumocephalus and its time course generally seems to be more important than the mere volume when assessing fitness to fly.

## Data Availability

Not applicable.
